# Dimethylfumarate protects against TNF-α-induced secretion of inflammatory cytokines in human endothelial cells

**DOI:** 10.1186/s12950-015-0094-z

**Published:** 2015-08-06

**Authors:** Simon Gerhardt, Veronika König, Monika Doll, Tsige Hailemariam-Jahn, Igor Hrgovic, Nadja Zöller, Roland Kaufmann, Stefan Kippenberger, Markus Meissner

**Affiliations:** Department of Dermatology, Venereology and Allergology, Goethe-University, Theodor-Stern-Kai 7, D-60590 Frankfurt am Main, Germany; Department of Cardiology, Gutenberg-University, Mainz, Germany

**Keywords:** Dimethylfumarate, p65, MCP-1, TNF-α, ΙκΒα

## Abstract

**Background:**

Inflammation, angiogenesis and oxidative stress have been implicated in the pathogenesis of various vascular diseases. Recent evidence suggests that dimethylfumarate (DMF), an antiposriatic and anti-multiple sclerosis agent, possesses anti-inflammatory, anti-oxidative and anti-angiogenic properties. Here, we analyze the influence of DMF on TNF-α-induced expression of the important pro-inflammatory and pro-atherogenic chemokine MCP-1 and investigate the underlying mechanisms of this expression.

**Findings:**

We analyzed constitutive and TNF-α-induced expression of MCP-1 in human umbilical vascular endothelial cells (HUVEC) +/− DMF treatment via enzyme-linkes immunosorbent assay (ELISA). DMF significantly inhibited the protein expression levels in a time- and concentration-dependent manner. Furthermore, MCP-1 mRNA expression was also reduced in response to DMF, as demonstrated by RT-PCR. Thus, the regulation occurs at the transcriptional level. Interestingly, DMF prolonged the TNF-α-induced p38 and JNK phosphorylation in HUVEC, as demonstrated by Western blot analysis; however, the p38 and JNK inhibitor SB203580 did not affect the DMF-conveyed suppression of TNF-α-induced MCP-1 expression. DMF suppressed the TNF-α-induced nuclear translocation and phosphorylation (Serine 536) of p65 in these cells. These results were additionally approved by p65 luciferase promoter assays. Furthermore, we found that DMF slightly inhibited the early degradation of IκBα. In addition, we verified our results using other important inflammatory cytokines such as CCL-5, PDGF-BB, GM-CSF and IL-6.

**Conclusion:**

DMF suppresses various TNF-α-induced pro-inflammatory and pro-atherogenic cytokines/chemokines in human endothelial cells. This action is regulated by reduced p65 activity and nuclear translocation, which can be explained in part by the reduced early degradation of IκBα and more important the reduced phosphorylation of p65 at Serine 536. These effects were independent of the p38, PI3K and p42/44 signaling pathways. As a result, DMF might be suitable for treating patients with vascular diseases.

**Electronic supplementary material:**

The online version of this article (doi:10.1186/s12950-015-0094-z) contains supplementary material, which is available to authorized users.

## Findings

### Introduction

The association between inflammation and angiogenesis in pathological conditions is widely accepted. There are many chronic inflammatory diseases – for example, psoriasis, diabetes, atherosclerosis and cancer – in which inflammation and angiogenesis are dependent on each other. Therefore, inflammation and angiogenesis are two processes that develop coordinately [1]. Activated endothelial cells are important sources of pro-inflammatory and pro-atherogenic cytokines and chemokines [2]. Chemokines such as MCP-1 [3], GM-CSF [4], PDGF-BB [5], IL-6 [6] and CCL-5 [7] are known to promote atherosclerosis and myocardial ischemia [3, 4]. Therefore, medications suppressing angiogenesis as well as the expression of pro-inflammatory cytokines and chemokines are promising candidates for the treatment and prevention of chronic inflammatory diseases.

Dimethylfumarate (DMF) has been successfully used in the treatment of psoriasis, a chronic inflammatory disease, for more than 40 years [5], and several clinical trials have proven its efficacy. Last year, DMF was approved for the treatment of relapsing-remittent multiple sclerosis (MS) [6]. There are a number of mechanisms by which DMF mediates its anti-inflammatory action. Stoof et al. showed that Il-8, Gro alpha, IP-10 and Mig are downregulated during DMF treatment [7]. In addition, DMF treatment has been shown to result in leukocytopenia and a reduction in the number of CD8+ and CD4+ T cells in psoriasis lesions, primarily due to apoptosis [8, 9]. Recent research has revealed the anti-oxidant properties of DMF in various conditions induced in part by the expression of anti-oxidant molecules such as HO-1 and the activation of Nrf-2 [10]. Vandermeeren et al. demonstrated that DMF is an effective inhibitor of cytokine-induced VCAM-1, ICAM-1 and E-selectin expression in human endothelial cells [11]. Additional investigations revealed that DMF inhibits nuclear translocation of activated NF-κB in endothelial cells and suppresses the activation of NF-κB in human dendritic cells [12, 13]. Recently, we demonstrated that DMF inhibits angiogenesis via the downregulation of VEGFR-2 expression [14]. These results were the first hint that DMF also possesses distinct anti-angiogenic actions [15]. Recently, it was demonstrated that DMF might be a therapeutic drug for patients with vascular diseases. Oh et al. demonstrated that DMF attenuates restenosis in a rat carotid artery balloon injury model [16]. Silhavy et al. also showed that DMF blocks the pro-inflammatory actions of human CRP in transgenic spontaneously hypertensive rats [17]. Ashafrian et al. demonstrated the cardioprotective action of DMF in an *in vivo* model of myocardial infarction [18]. In addition, Milenkovic et al. demonstrated that DMF has beneficial effects in autoimmune myocarditis [19].

Here, we investigated whether DMF suppresses the constitutive and TNF-α-induced expression of important pro-inflammatory chemokines and cytokines in human endothelial cells. We also analyzed the underlying mechanisms of this expression.

## Material and methods

### Reagents

Recombinant human TNFα was purchased from R&D Systems (Minneapolis,MN, USA). Dimethylfumarate, SB203580, PD98059 and Wortmannin were obtained from Sigma-Aldrich (Hamburg, Germany).

### Cell culture

Human umbilical vascular endothelial cells (HUVECs) were purchased from PromoCell (Heidelberg, Germany) and were cultured until the fifth passage at 37 °C and 5 % CO_2_ in Endothelial Cell Growth Medium (Lonza, East Rutherford, NJ, USA).

### Enzyme-linked immunosorbent assay (ELISA)

The concentrations of MCP-1, PDGF-BB, GM-CSF, CCL-5 and IL-6 in cell culture supernatants were determined by enzyme-linked immunosorbent assay (ELISA). Commercially available ELISA kits (R&D Systems, Minneapolis, MN, USA) were used for the quantification as described in the manufacturer’s instructions.

### Western blot analysis

The protein extracts were prepared as described previously [14]. Following SDS-PAGE and electroblotting, the membranes were incubated with the following primary antibodies: p65, phospho-p65 (Ser536), IκBα p38, p42/44, JNK, pospho-p38, phospho-p42/44, phospho-JNK, Phospho-Akt, Akt and tubulin (Cell Signaling, Danvers, MA, USA); Laminin A/B (Santa Cruz, Dallas, Texas, USA); SP1 (Sigma-Aldrich, St. Louis, MO, USA). Primary antibody application was followed by incubation with horseradish peroxidase-conjugated secondary antibodies (anti-mouse and anti-rabbit IgG, Amersham, Uppsala, Sweden; anti-goat, Dako, Glostrup, Denmark). The blots were visualized using an enhanced chemiluminescence detection system (ECL) (Amersham, Freiburg, Germany) according to the manufacturer’s instructions. Densitometry was used to quantify band intensities using ImageJ (v1.29 s). Optical densities of the bands were corrected for loading differences based on corresponding control bands.

### RNA extraction and RT-PCR

The RT-PCR analyses were performed using total RNA (150 ng) extracted from sub-confluent cell cultures. The total cellular mRNA was isolated using the RNeasy Mini Procedure (Qiagen, Hilden, Germany) after DNase digestion. The RT-PCR analyses for MCP-1 and GAPDH were performed with a One Step RT-PCR Kit (Qiagen, Hilden, Germany). The PCR products were resolved by gel electrophoresis in a 1-2 % agarose gel, and the ethidium bromide-stained bands were visualized using an ultraviolet transilluminator. The primer sets for MCP-1 and GAPDH were previously published [14].

### Transient transfection and analysis of reporter gene expression

HUVECs (1.0 × 10^5^ cells/well in 12-well plates) were transfected with 0.5 μg of the appropriate *firefly* luciferase construct and 0.1 μg phRG-TK vector (Promega, Madison, WI, USA) using the SuperFect transfection reagent (Qiagen). The P TransLucent NfkB promoter vector was purchased from Panomics (Affymetrix, Santa Clara, CA, USA). Twenty-four hours after transfection, the cells were treated with vehicle (0.3 % DMSO) or with DMF for 24 h. The luciferase activity was measured using a Dual-Luciferase Reporter Assay System (Promega, Madison, WI, USA).

### Fluorescence microscopy

HUVECs were plated onto 8-well chamber slides (Lab-Tek, Christchurch, New Zealand) at a density of 80 %/well. The cells were grown in endothelial cell basal medium with 5 % FCS for 24 h, fixed in 4 % paraformaldehyde at room temperature for 10 min, and permeabilized with 0.1 % Triton X-100 for 5 min at room temperature. Permeabilized cells were rinsed three times with PBS and incubated in blocking solution (1 % bovine serum albumin/PBS) for 30 min at room temperature to remove nonspecific binding of the antibody. All subsequent steps were carried out at room temperature, and cells were rinsed three times in 1 % bovine serum albumin/PBS between each of the steps. p65 was detected using a rabbit polyclonal anti-NfkB p65 antibody (D14E12; Cell Signaling) and an Alexa Fluor 594-conjugated goat anti-rabbit IgG secondary antibody (Alexa). The cytoskeleton was stained by phalloidin (Alexa-Fluor 488). The slides were mounted in Vectashield immunofluorescence mouting medium (Vector Laboratories) and viewed with fluorescence microscopy. Cells were counterstained for nuclei with Hoechst staining.

### Statistical analysis

The data are expressed as the mean ± SEM of at least three independent experiments. The statistical analyses were performed using Student’s *t*-test.

## Results

### Dimethylfumarate suppresses constitutive and TNF-α-induced MCP-1 protein expression

**Fig. 1:**
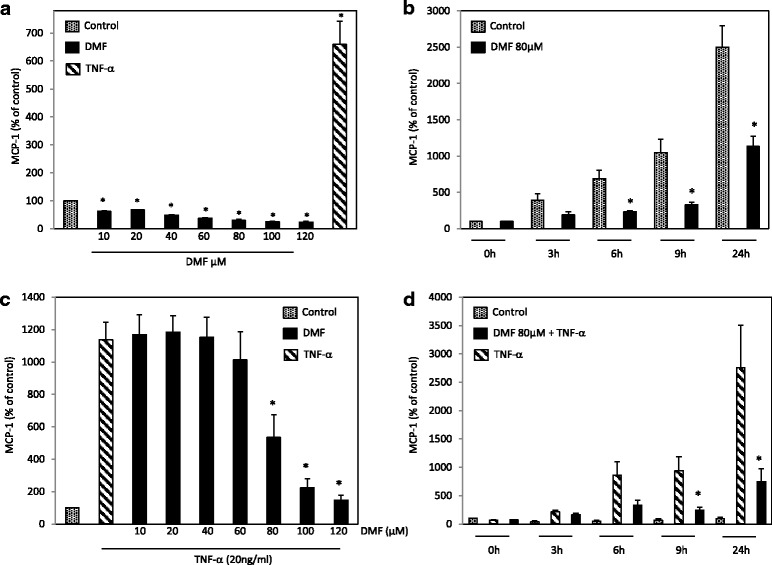
Analysis of constitutive and TNF-α-induced MCP-1 expression during the treatment with DMF in HUVECs. We assayed the MCP-1 protein content in culture supernatants by MCP-1 ELISA according to the manufacturer’s instructions. **a** HUVECs were mock-treated (solvent only) or treated with DMF at the indicated concentrations for 24 h. **b** HUVECs were mock-treated (solvent only) or treated with DMF (80 μM) for the indicated times. **c** HUVECs were mock-treated (solvent only), treated with TNF-α (20 ng/ml) or DMF at the indicated concentrations + TNF-α for 24 h. **d** HUVECs were mock-treated (solvent only) or treated with TNF-α (20 ng/ml) or DMF (80 μM) + TNF-α for the indicated times. The mean values from three triplicate experiments are presented as the mean ± SEM. We analyzed the data using the Student’s *t*-test. *p < 0.05

First, we analyzed the effect of DMF on constitutive MCP-1 expression in HUVECs. DMF inhibited MCP-1 expression significantly in a time- and concentration-dependent manner (Fig. 1a,b). The concentrations and incubation times that we adopted have been published recently and have been proven to have no cytotoxic effects in HUVEC [14]. DMF in concentrations less than 20 μM reduced the MCP-1 expression level by almost 50 %; within 3 h of treatment, the MCP-1 expression was significantly reduced compared with that of the matching controls. To determine whether DMF was also able to reduce TNF-α-induced MCP-1 expression, we analyzed the effects of the combinational treatment with DMF and TNF-α (20 ng/ml) in a time- and concentration-dependent manner. We found that 80 μM DMF reduced the TNF-α-induced MCP-1 expression by more than 50 % (Fig. 1c). A first significant reduction of MCP-1 expression could be observed as early as 6 h after treatment with 80 μM DMF and TNF-α (Fig. 1d).

### Dimethylfumarate inhibits constitutive and TNF-α-induced MCP-1 mRNA expression

**Fig. 2:**
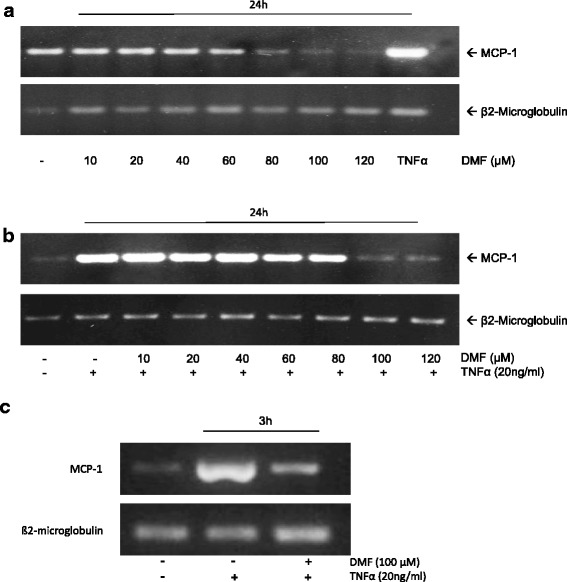
Analysis of constitutive and TNF-α-induced MCP-1 mRNA expression during the treatment with DMF in HUVECs. We isoalted total cellular mRNA and performed RT-PCR analyses for MCP-1 and β2-microglobulin. **a** HUVECs were mock-treated (solvent only) or treated with DMF at the indicated concentrations for 24 h. **b** HUVECs were mock-treated (solvent only) or treated with TNF-α (20 ng/ml) or DMF at the indicated concentrations + TNF-α for 24 h. **c** HUVECs were mock-treated (solvent only) or treated with TNF-α (20 ng/ml) or DMF (100 μM) + TNF-α for 3 h. The experiments were performed with comparable results at least 5 times

To determine whether a transcriptional mechanism was responsible for the reduced MCP-1 expression observed in response to DMF, we performed RT-PCR analysis. As observed in our protein expression analysis, DMF inhibited the constitutive as well as the TNF-α-induced MCP-1 mRNA expression after 24 h (Fig. 2a,b). To exclude that this effect did not only occur at later time points, we also performed RT-PCR analysis after 3 h of treatment (Fig. 2c). We were able to demonstrate that DMF supresses TNF-α induced MCP-1 mRNA expression effectively.

### Dimethylfumarate prolongs p38 and JNK phosphorylation in TNF-α-treated HUVECs

**Fig. 3:**
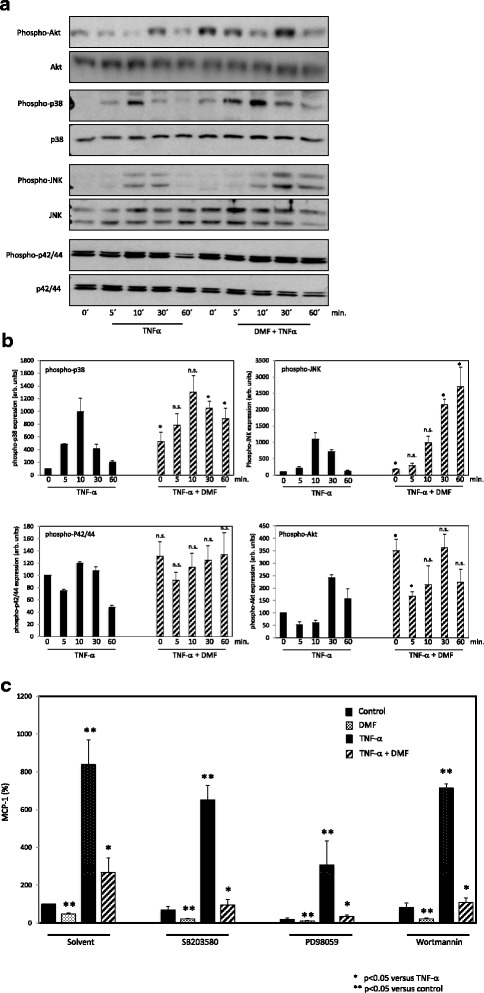
Analysis of the effects of DMF on p38, JNK, Akt and p42/44 phosphorylation in TNF-α-treated HUVECs and the influence on MCP-1 expression. **a** p38, JNK, Akt and p42/44 expression and phosphorylation: Western blot analysis of HUVECs treated with vehicle (solvent only), TNF-α (20 ng/ml) or DMF (80 μM, 3 h pre-treatment) + TNFα for 60 min. **b** Densitometry analysis: The results were normalized to the expression of the nonposphorylated controls (p38, JNK, AKT, p42/44). The relative expression of the posphorylated protein is presented in arbitrary units (arb. units). The mean values from at least three independent experiments are presented as the mean ± standard deviation. The TNF-α only treated bands served as control to the combinational treatment (DMF+ TNF-α). We analyzed theata using the Student’s *t* test. *p <0.05; n.s. not significant. **c** Signaling pathway blockade: MCP-1 ELISA of HUVECs treated with vehicle (solvent only), TNF-α (20 ng/ml) or DMF (80 μM, 3 h pre-treatment) + TNF-α for 60 min. We performed the blockade of the signaling pathways via treatment with SB203580 (1 μM), Wortmannin (5 μM) and PD 98059 (30 μM) 30 min before the main treatment. We present the mean values from three triplicate experiments as the mean ± SEM. We analyzed the data using the Student’s *t*-test. *p < 0.05 versus TNF-α; **p < 0.05 versus control

The p38/JNK, PI3K/Akt and p42/44 signaling pathways are known to be important in TNF-α-induced MCP-1 expression. Therefore, we analyzed the phosphorylation status of p38, JNK, Akt and p42/44 in HUVEC over time (0–60 min.) in response to TNF-α (20 ng/ml) or TNF-α plus DMF (80 μM, 3 h pre-treatment). Surprisingly, we found that DMF prolonged the TNF-α-induced phosphorylation of p38 and JNK in HUVECs significantly based on Western blot and densitometry analyses (Fig. 3a,b). For Akt phosphorylation, we observed an early significant (0 and 5 min.) superinduction but no prolonged phosphorylation compared with TNF-α treatment. The posphorylation of p42/44 was not influenced by DMF treatment. To determine whether these signaling pathways were responsible for the effects of DMF on MCP-1 expression, we performed an enzyme-linked immunosorbent assay (ELISA) with the p38/JNK inhibitor SB203580, the PI3K/Akt inhibitor Wortmannin and the p42/44 inhibitor PD98059. Interestingly, blocking these signaling pathyways did not affect the DMF-induced suppression of MCP-1 expression (Fig. 3c).

### Dimethylfumarate reduces TNF-α-induced p65 nuclear entry, phosphorylation and promoter activity and inhibites IκBα degradation

**Fig. 4:**
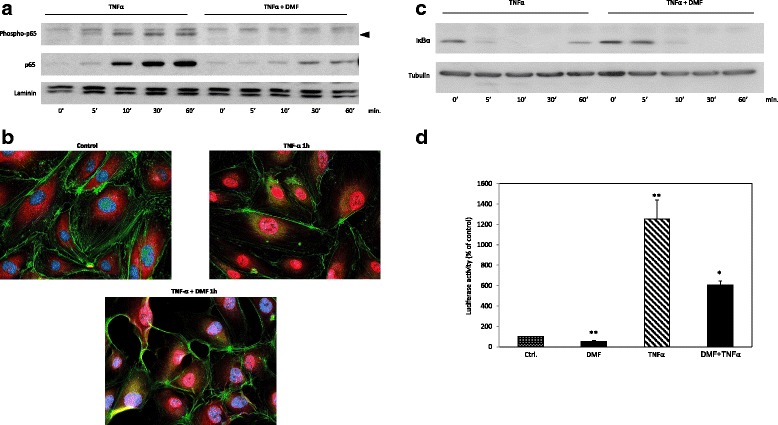
Analysis of TNF-α-induced p65 nuclear entry, phosphorylation (Ser 536), promoter activity and IκBα degradation during DMF treatment. **a** Nuclear p65 translocation and phosphorylation: Western blot analysis of nuclear proteins of HUVECs treated with vehicle (solvent only), TNF-α (20 ng/ml) or DMF (80 μM, 3-h pre-treatment) + TNF-α for 60 min. The phosphorylated p65 (Serine 536) band is marked by an arrow head. **b** Representative immunofluorescent analysis of p65 in HUVECs that were treated with vehicle, TNF-α (20 ng/ml) or DMF (80 μM, 3 h pre-treatment) + TNF-α for 1 h. **c** IkB degradation: Western blot analysis cytosolic proteins of HUVECs treated with vehicle (solvent only), TNF-α (20 ng/ml) or DMF (80 μM, 3 h pre-treatment) + TNF-α for 60 min. **d** Analyses of the NfκB luciferase (Luc) reporter constructs in HUVECs treated with vehicle (solvent only), DMF (80 μM), TNF-α (20 ng/ml) or DMF (80 μM) + TNF-α for 24 h, respectively. The Luc activities are expressed as relative luciferase activity as a percent (mean ± SEM of at least five independent triplicate assays). *p < 0.05 versus TNF-α; **p < 0.05 versus control

It is well known that TNF-α mediates its effects, such as increased MCP-1 expression, mainly by nuclear translocation and phosphorylation of p65 after the degradation of IκBα. Recently, Loewe et al. demonstrated the reduced nuclear translocation of p65 in response to DMF treatment in HUVEC, partially reversing the TNF-α effects. In our experiments, we observed reduced p65 translocation but additionally recorded reduced nuclear p65 phosphorylation at Serine 536 during DMF treatment (Fig. 4a). Furthermore, we found a significant reduction in nuclear translocation of p65 by immunofluorescence staining of NFκB p65 in HUVECs treated with a combination of DMF and TNF-α compared with a treatment of TNF-α alone (Fig. 4b). To exclude a reduced overall expression of p65 during the treatment with DMF, we analyzed p65 expression by Western blot analysis (Additional file 1); no reduction of p65 expression was observed.

In contrast with Loewe et al., we did observe a slightly reduced degradation of IκBα in response to treatment with DMF, which might in part explain the early (5-30 min) decreased p65 nuclear translocation (Fig. 4c). In HUEVCs transiently transfected with an NfκB-luciferase promoter construct, DMF significantly reduced both the basal transcriptional activity as well as the TNF-α-dependent NfκB promoter activity after 24 h of treatment (Fig. 4d).

### Dimethylfumarate effectively suppresses the TNF-α-induced expression of CCL-5, PDGF-BB, GM-CSF and IL-6

**Fig. 5:**
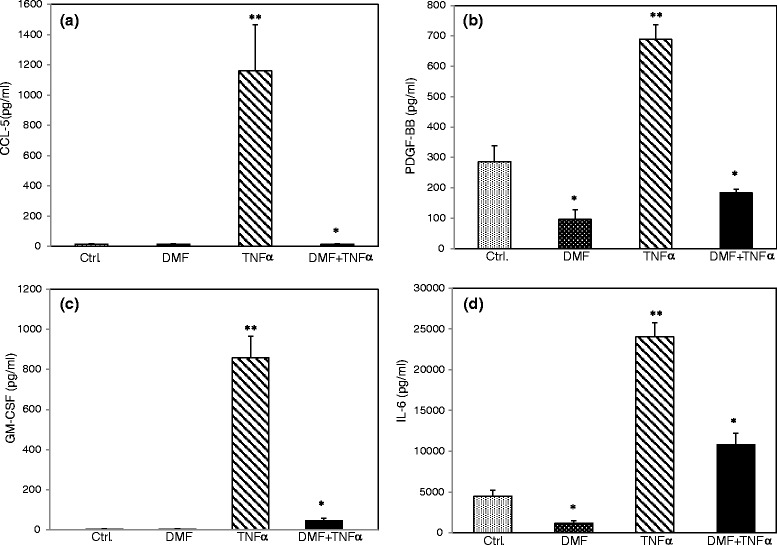
Analysis of constitutive and TNF-α-induced expression of various inflammatory cytokines/chemokines during the treatment with DMF in HUVECs. We assayed the protein content in culture supernatants by ELISA (R&D Systems, Minneapolis, US according to the manufacturer’s instructions. The HUVECs cells were left untreated (solvent only, DMSO 0.1 %) or treated with TNF-α (20 ng/ml), DMF (80 μM) and DMF + TNF-α for 24 h. **a** CCL-5; (**b**) PDGF-BB; (**c**) GM-CSF; (**d**) IL-6. The mean values from three triplicate experiments are presented as the mean ± SEM. We analyzed the data using the Student’s *t*-test. *p < 0.05 versus TNF-α; **p < 0.05 versus control

To further analyze whether DMF suppresses other important pro-inflammatory and pro-atherogen cyto- and chemokines in human endothelial cells, we examined the expression of CCL-5 (RANTES), PDGF-BB, GM-CSF and IL-6 using an ELISA. We treated the HUVECs with DMF (80 μM), TNF-α (20 ng/ml), and a combination of TNF-α and DMF or the solvent alone for 24 h. The TNF-α-induced expression levels of GM-CSF and IL-6 were significantly reduced, and the TNF-α-induced expression levels of CCL-5 and PDGF-BB were abolished completely (Fig. 5a–d).

## Discussion

The endothelial cell is one of the central components of various inflammatory diseases such as psoriasis, diabetes, cancer and rheumatoid arthritis [20]. Recent evidence has shown that chronic inflammation is associated with accelerated atherosclerosis and increased cardiovascular mortality and morbidity [21]. It is believed that the therapy targeting the inflammatory processes shared in psoriasis and atherosclerosis may reduce cardiovascular risk in psoriatic patients [22]. In our study, we analyzed the influence of DMF, a potent anti-psoriatic and anti-MS drug, on the constitutive and TNF-α-induced expression of MCP-1 and other important pro-inflammatory and pro-atherogenic cyto- and chemokines. We additionally examined the underlying mechanisms of this expression. We demonstrated that DMF significantly suppressed the constitutive and TNF-α-induced expression of MCP-1, CCL-5, PDGF-BB, GM-CSF and IL-6 in HUVEC. Furthermore, DMF eliminated the TNF-α-induced expression of CCL-5 and PDGF-BB. Recently, Seidel et al. demonstrated that DMF suppresses TNF-α-induced CCL-5 and IL-6 expression in smooth muscle cells via reduced p65 nuclear entry [23]. Our findings not only confirm these observations in another cell type involved in atherogenesis but also broaden these results regarding the chemokines MCP-1, PDGF-BB and GM-CSF. The TNF-α-induced expression of these pro-inflammatory cyto- and chemokines is known to be mainly transcriptionally regulated by NfκB [24]. We used MCP-1 as a model chemokine and demonstrated a transcriptional way of regulation. To determine whether this regulation was controlled by the p38/JNK, PI3K/Akt or p42/44 signaling pathways, we performed Western blot analysis. We detected prolonged and superinduced posphorylation of p38 and JNK in response to DMF and TNF-α compared with TNF-α alone. Interestingly, Seidel et al. demonstrated an enhancement of PDGF-BB-induced p38-phosphorylation by DMF in smooth muscle cells [25]. On the other hand, Gesser et al. found that DMF did not influence IL-1ß-induced p38-phosphorylation in human keratinocytes [26]. Recently, Xie et al.demonstrated that DMF induces p38, JNK and p42/44 phosphorylation in colon carcinoma cell lines and induces necroptosis [27]. In our experiments, we did not find any significant influence of DMF on p42/44 phosphorylation. Therefore, this effect might be cytokine- and/or cell-type specific. To determine whether the signaling pathways were related to the inhibition of TNF-α-induced MCP-1 expression, we blocked them using specific inhibitors. Interestingly, blocking the p38, JNK, Akt and p42/44 pathways did not affect the DMF-induced MCP-1 suppression. Therefore, we showed that the prolonged activity of p38 and JNK by DMF treatment was independent of its effect on MCP-1 expression.

It is well known that TNF-α is a potent NfkB inductor and that MCP-1 and the other analyzed cyto- and chemokines are regulated by NfκB nuclear translocation and phosphorylation [28]. Therefore, we studied the TNF-α-induced nuclear translocation and phosphorylation of p65 to determine whether DMF has the ability to reduce these effects. We found that DMF clearly inhibits the TNF-α-induced nuclear p65 translocation. This phenomenon has also been described by Loewe et al. [13]. We extended this observation concerning the phosphorylation status of p65 by demonstrating that the TNF-α-induced p65 phosphorylation was almost completely eliminated by DMF. In addition, we found that treatments with DMF resulted in slightly reduced early degradation of IκBα . This observation only explains in part the early decreased p65 nuclear translocation (5-30 min). The later inhibition of p65 translocation by DMF seems to be independent of IκBα degradation, as demonstrated by Peng et al. in dendritic cells treated with DMF [29]. Sasaki et al. demonstrated that phosphorylation of p65 on Serine 536 defines an IκBα-independent NF-κB pathway [30]. This group suggests that there are two mutually exclusive populations of p65 that are distinguished in their phosphorylation status at serine 536. They postualte that during activation, the populations translocate independently and induce transcription of different genes. These results are in accordance with our experiments demonstrating a significant reduction of p65 phosphorylation at Serine 536, which now might explain the reduced p65 translocation mainly independent of IκBα in human endothelial cells. Thus, our results not only confirm but also broaden and explain the results demonstrated by Loewe et al. concerning the nuclear entry of p65 [13].

To prove that the DMF-conveyed reduced nuclear translocation and phosphorylation of p65 is functionally relevant, we performed NFkB luciferase promoter assays demonstrating significant inhibition of constitutive and TNF-α-induced promoter activity by DMF.

In summary, we demonstrated that DMF inhibits constitutive and TNF-α-induced expression of pro-inflammatory and pro-atherogenic cytokines in human endothelial cells. Our findings are consistent with the inhibition of nuclear translocation, phosphorylation and activity of p65, which can be explained in part by the reduced early degradation of IκBα and more important the reduced phosphorylation of p65 at Serine 536. These results, which may help to explain the cardioprotective and anti-inflammatory effects of DMF, highlight its potential importance in the future treatment of vascular diseases.

## Additional file

Additional file 1: Figure S1. Analysis of p65 expression during treatment with DMF, TNF-α and DMF + TNF-α in HUVECs. Western blot analysis of whole lysate proteins of HUVECs treated with vehicle (solvent only), TNF-α (20 ng/ml) or DMF (80 μM) + TNF-α for the indicated times. The experiments were performed with comparable results at least 3 times. (PPTX 1310 kb)

## Abbreviations

HUVEC: human umbilical vacular endothelial cells
DMF: Dimethylfumarate
DMSO: Dimethylsulfoxide
TNF: Tumor necrosis factor
MCP: Monocyte chemoattractant protein
IL: interleukine
CCL: Chemokine ligand
