# Supplementary figures and images for: Dimethylfumarate protects against TNF-α-induced secretion of inflammatory cytokines in human endothelial cells
Source: J Inflamm (Lond). 2015 Aug 6;12:49. doi: 10.1186/s12950-015-0094-z (PMC4525722; doi:10.1186/s12950-015-0094-z)

## Slide 1
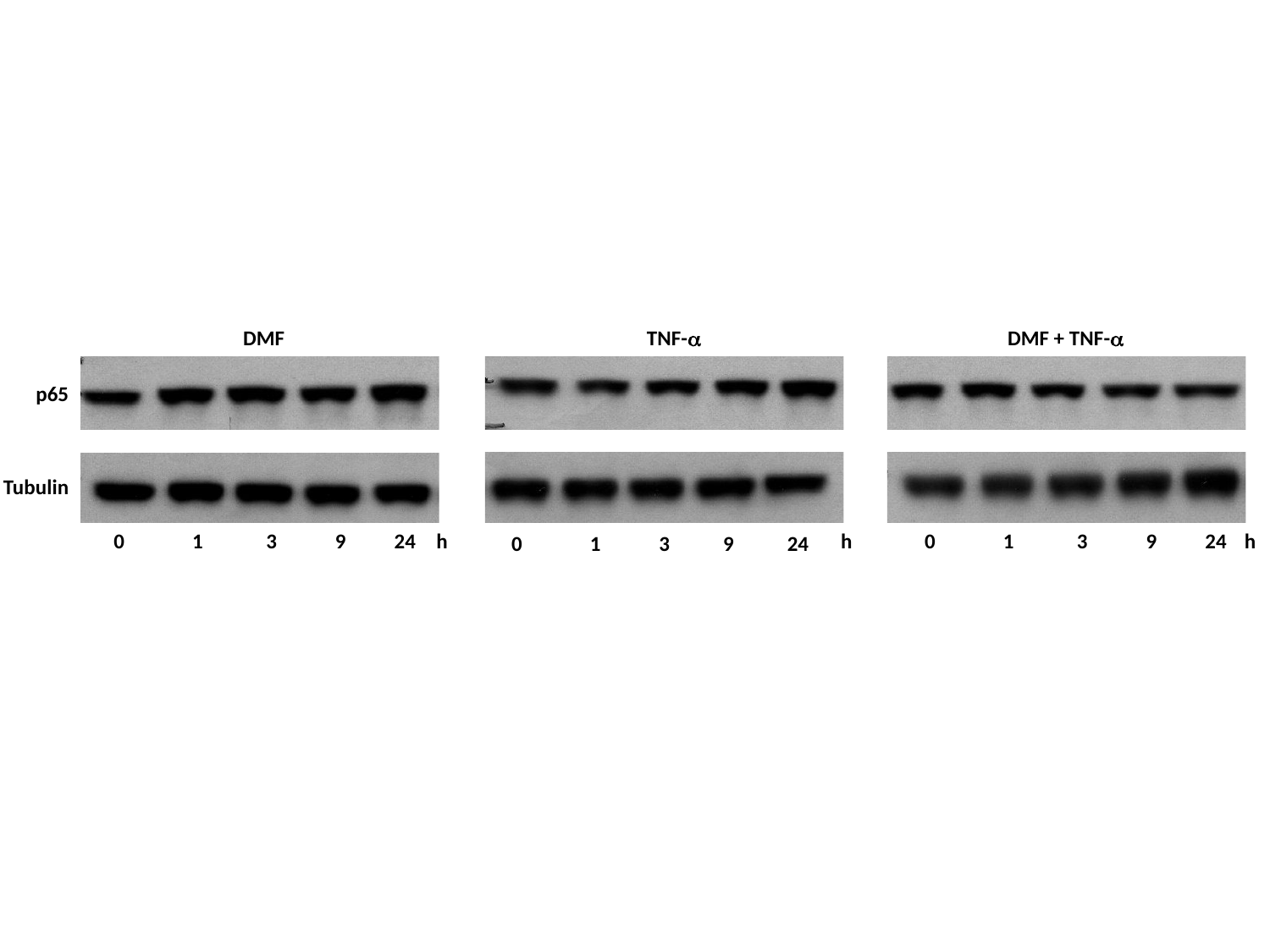

DMF
TNF-a
DMF + TNF-a
0 1 3 9 24
h
h
0 1 3 9 24
h
0 1 3 9 24
p65
Tubulin

Supplement: Additional file 1: Figure S1. — Analysis of p65 expression during treatment with DMF, TNF-α and DMF + TNF-α in HUVECs. Western blot analysis of whole lysate proteins of HUVECs treated with vehicle (solvent only), TNF-α (20 ng/ml) or DMF (80 μM) + TNF-α for the indicated times. The experiments were performed with comparable results at least 3 times. (PPTX 1310 kb) [file 12950_2015_94_MOESM1_ESM.pptx]
